# 30 years of media coverage on high drug prices in the US – a never-ending story or a time for change?

**DOI:** 10.1186/2052-3211-8-S1-P13

**Published:** 2015-10-05

**Authors:** Christine Leopold, James D Chambers, Anita K Wagner

**Affiliations:** 1 Department of Population Medicine, Harvard Medical School and Harvard Pilgrim Health Care Institute, Boston, Massachusetts, 02215, USA; 2 Center for the Evaluation of Value and Risk in Health, Institute for Clinical Research and Health Policy Studies, Tufts Medical Center, Boston, Massachusetts, 02116, USA

## Background

US drug prices are among the highest worldwide as US policy makers have historically been reluctant to embrace price regulations, instead relying on market forces to set prices. However, the introduction of a number of breakthrough, highly effective and high-cost specialty medicines over the past years has stoked the fire of the long-running drug price debate in the USA. The prices of those specialty medicines – more than $100,000 per treatment course – have resulted in widespread outcry among patients, providers, insurers, and members of the Congress and the Senate. We aimed at analyzing whether the recent debate on drug prices reflects a sign of change in the drug pricing debate in US print media.

## Methods

We used LexisNexis Academia – a database of legal, news and business sources – to determine how frequently the *New York Times* and *Wall Street Journal* featured articles including the term ‘drug pricing’ from January 1985 through June 2015. For the purpose of analyzing the media releases, we included each article in either one of the four categories: increase of drug prices, innovation, stakeholder's response and proposed solutions and described the change of debate in each category.

## Results

The media search on ‘drug pricing’ over the last 30 years showed that facts around high-cost medicines in the USA are changing: Drug prices of on- and off-patent medicine increase rapidly but from launch prices that are orders of magnitude higher than in the past[1]. Some new products are breakthrough therapies rather than marginal improvements over existing treatments with an indication for millions of patients with steep prices. Consequently more and more stakeholders (like doctors, public and private payers as well as Senators) are taking action and questioning whether the USA should contain free pricing for prescription medicines[2].

## Conclusions

The frequency and content of media reports on drug prices in the *New York Times* and *Wall Street Journal* in the past 30 years may indicate a time for legal and policy change.
